# Systematic review and meta-analysis of diagnostic accuracy of one-step nucleic acid amplification for lymph node metastases of papillary thyroid carcinoma

**DOI:** 10.1007/s00423-025-03742-4

**Published:** 2025-06-11

**Authors:** Maria Magdalena Llompart-Coll, Paula Domínguez-Garijo, Martí Manyalich-Blasi, Gemma Domènech-Gómez, Iria Perales-Galan, David Saavedra-Pérez, Maria Teresa Rodrigo, Sergi Vidal-Sicart, Miguel Pera-Roman, Oscar Vidal-Pérez

**Affiliations:** 1 Department of General and Digestive Surgery, Hospital Clínic Barcelona - ICMDM, Universitat de Barcelona, Barcelona, Spain; 2 Department of Medical Statistics, Hospital Clínic Barcelona, Universitat de Barcelona, Barcelona, Spain; 3 Department of Pathology, Hospital Clínic Barcelona - CBD, Universitat de Barcelona, Barcelona, Spain; 4 Department of Nuclear Medicine, Hospital Clínic Barcelona - CDI, Universitat de Barcelona, Barcelona, Spain; 5 Institut d’Investigacions Biomèdiques August Pi i Sunyer (IDIBAPS), Universitat de Barcelona, Barcelona, Spain

**Keywords:** Systematic review, Meta-analysis, Nucleic acid amplification techniques, Lymphatic metastasis, Carcinoma, papillary, Thyroid neoplasms

## Abstract

**Purpose:**

To evaluate the diagnostic accuracy and detection rate of lymph node metastases (LNM) in Papillary Thyroid Carcinoma (PTC) using the One-Step Nucleic Acid Amplification (OSNA) technique compared to conventional pathological methods.

**Methods:**

A systematic search was conducted in PubMed, The Cochrane Library, Scopus, and Web of Science from May 1, 2023, to June 30, 2023, for studies published from January 1, 2005, to April 30, 2023. Observational studies assessing OSNA for LNM in PTC patients were included. Two independent reviewers performed study selection, record screening, data extraction, and quality assessment using QUADAS-2. Meta-analysis was conducted with Posit software (RStudio).

**Results:**

Seven studies (2014–2021) involving 1,424 lymph nodes from 207 PTC patients were analyzed. The pooled sensitivity, specificity, and AUC of OSNA for detecting LNM were 0.905 (95% CI 0.838–0.946), 0.884 (95% CI 0.834–0.921), and 0.848, respectively.

**Discussion:**

OSNA shows promising diagnostic accuracy compared to conventional pathological methods, with the potential to improve real-time lymphatic staging and aid intraoperative decision-making. Limitations include study heterogeneity and a lack of randomized controlled trials, affecting generalizability. Further research is needed to validate the long-term benefits of OSNA in clinical practice.

**Supplementary Information:**

The online version contains supplementary material available at 10.1007/s00423-025-03742-4.

## Introduction

Papillary thyroid carcinoma (PTC) is the most common endocrine malignancy, representing more than 80% of all thyroid cancers [1]. Its dissemination pattern tends to spread to regional lymph nodes, being the central neck compartment the most commonly involved [2]. The presence of cervical lymph node metastases at diagnosis is the most important factor of local recurrence, significantly increasing morbidity and mortality [3]. The American Thyroid Association (ATA) [4] only recommends central compartment neck dissection in cases with clinically involved cervical neck nodes or advanced primary tumors (T3 or T4), and performed by experienced surgeons due to the high risk of morbidity of the technique. Unfortunately, clinical examination and neck ultrasound may present false-negative rates of up to 30% due to occult cervical lymph node metastases [5].

Selective sentinel lymph node biopsy (SLNB) has emerged as an effective technique for assessing intraoperatively the potential lymph node involvement [6]. Isotopic radio-guided SLNB has shown promising results when managing PTC surgical treatment [7].

However, when compared to definitive postoperative histological examination, intraoperative lymph node metastasis (LNM) assessment techniques resulted in false-negative rates between 12 and 15%, especially if small volume nodes or micrometastases were present [8].

PTC cells, both primary and metastatic, express Cytokeratin 19 mRNA, which should not be detected in unaltered/unaffected lymph nodes. CK19 is an intermediate filament protein commonly expressed in epithelial cells, including those of the thyroid gland. In malignancies such as PTC, CK19 is frequently overexpressed, making it a valuable biomarker for detecting metastatic spread. The presence of CK19 mRNA in lymph nodes indicates infiltration by epithelial-derived tumor cells, and its detection provides a molecular basis for identifying occult metastases [9].

The one-step nucleic acid amplification (OSNA) technique has emerged in recent years as a sensitive and rapid method to quantify CK19 mRNA intraoperatively. OSNA has shown significant success in breast cancer, where it is already incorporated into clinical guidelines in several countries. It offers high diagnostic performance, with a meta-analysis reporting pooled sensitivity and specificity of 87% and 92%, respectively [10]. OSNA’s accuracy in breast cancer is comparable to standard postoperative histopathology, but with the added benefit of enabling immediate surgical decisions. These findings suggest its potential applicability to other CK19-positive malignancies, such as PTC, particularly in improving the detection of metastatic lymph nodes [11, 12].

This study aims to determine the diagnostic accuracy and improved detection rate of PTC lymph node metastases by OSNA technique, compared to conventional pathological examination.

## Methods

This systematic investigation follows the protocols outlined by the Preferred Reporting Items for Systematic Reviews and Meta-Analyses (PRISMA) [13].

### Search Strategy

A detailed investigation was carried out within the timeframe of May 1 st, 2023, to June 30 th, 2023, across four key electronic bibliographic databases: PubMed, The Cochrane Library, Scopus, and Web of Science. To ensure the inclusion of the most recent evidence, a second search was conducted after completing the statistical analysis and prior to manuscript submission in May 2024, with no additional eligible studies identified. The search was targeted at identifying studies published from January 1 st of 2005 to April 30 th of 2023 and concerning the efficacy of the OSNA method in detecting LNM in patients diagnosed with PTC. The search was performed using the following keywords and search formulas: ‘Systematic Review’, ‘Meta-Analysis’, ‘Nucleic Acid Amplification Techniques’, ‘Lymphatic Metastasis’, ‘Carcinoma, Papillary’, and ‘Thyroid Neoplasms’. The search strategies were structured as: “One-Step Nucleic Acid Amplification” OR OSNA AND “Lymph Node” OR “Lymphatic” Metastasis AND “Papillary Thyroid” AND “Cancer OR Neoplasm OR Carcinoma.” These terms were adapted to the specific syntax of each database to ensure thorough and comprehensive search results. A thorough examination of the references in all chosen articles was conducted to find any supplementary studies.

### Inclusion and Exclusion Criteria

The included observational studies (case series, case-control studies, and cohort studies): (1) concerned individuals diagnosed with PTC (V-VI Bethesda criteria [14]); (2) assessed LNM with the OSNA technique, using the postoperative conventional histopathological analysis as the gold standard; (3) provided enough data to determine the counts of true positives, false positives, false negatives, and true negatives. No randomized trial or experimental studies were found. Studies concerning other types of cancer, missing standard histopathological data, literature not in English or Spanish, unpublished articles, reviews, conference summaries, letters, case narrations, or editorial commentary were excluded.

### Data extraction

Two separate reviewers, PDG and MMLC, independently performed the study selection, record examination, data collection, and quality assessment. Any discrepancies were evaluated and resolved by a third reviewer, MM. Compiled data included author name, publication date, study locale, methodological design, participant and lymph node counts, standard reference used methods, pre-surgical confirmation of CK19 expression via immunohistochemistry (IHC) after fine needle aspiration biopsy (FNAB) analysis, and the figures for true and false positives/negatives, including any nodes where findings did not align.

### Quality evaluation

Quality Assessment of Diagnostic Accuracy Studies (QUADAS-2) [15] was employed as a tool for addressing four principal quality domains in the different included studies: selection of patients, the test under scrutiny, the standard of comparison, and the sequence and timing of these components. Each section was meticulously scrutinized for bias and applicability concerns, with the exception of flow and timing, categorizing them into low, high, or uncertain risk levels.

### Data synthesis and statistical analysis

Statistical analysis was centrally performed by the Medical Statistics core facility (MedStats) of Fundació Clínic per a la Recerca Biomèdica (FCRB)- Institut D’Invetigacins Biomèdiques August Pi i Sunyer (IDIBAPS). Data from studies that provided the required comprehensive metrics to calculate Diagnostic Odds Ratios (DOR), as well as counts of true positives, false positives, false negatives, and true negatives were synthesized. These statistics, in addition to sensitivity and specificity, along with positive and negative predictive values, were collated. Heterogeneity was evaluated by means of the I2 statistic. For the meta-analysis, the number of cases and sample size in each group were used to compute the pooled Odds Ratios and their 95% confidence intervals (95% CI, that were estimated using the random-effects model described by Der Simonian and Laird [15] and the 0.5 correction in case of 0 events, with the metabin command of R. The same information was used to compute the pooled proportion (sensitivity and specificity) in each treatment group, using the same assumptions and the arcsine method with the metaprop command of R, and also to compute the likelihood ratio with the madauni command of R. The accuracy of the OSNA technique in comparison to standard histological methods was determined by analyzing the area under the curve of the summary receiver operating characteristic (SROC). The research did not include a subgroup analysis. All statistical analyses and figures were performed using Posit team (2024, RStudio: Integrated Development Environment for R. Posit Software, PBC, Boston, MA).

## Results

### Study selection

**Fig. 1:**
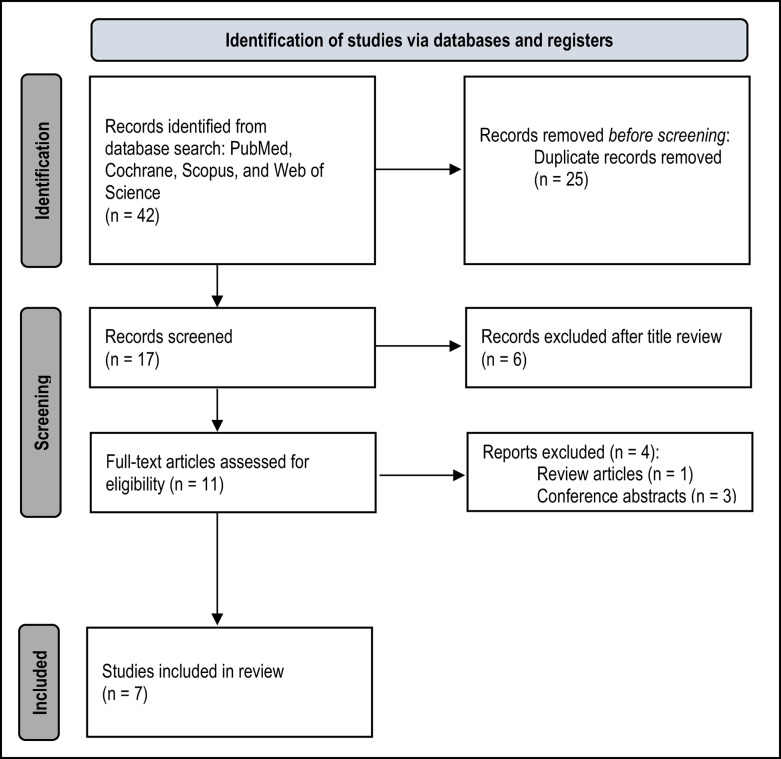
PRISMA 2020 flow diagram of the study selection process

The search strategy across PubMed, Cochrane, Scopus, and Web of Science yielded 42 records. After the exclusion of duplicates, 17 records remained, from which 6 were excluded due to the title, 3 for being conference abstracts [9, 16, 17] and 1 for being a review article [18]. Consequently, only 7 studies [19–23] met al.l the inclusion criteria and were incorporated into the qualitative and quantitative synthesis for this systematic review. Figure 1 illustrates diagram flow of the study selection process.

### Study characteristics

This meta-analysis evaluated seven studies published between 2014 and 2021, which together analyzed 1,424 lymph nodes from 207 patients diagnosed with PTC. The detailed characteristics of the included studies are given in Table 1. Each study compared standard histopathological examination with OSNA to detect PTC LNM. However, the approach to lymph node preparation varied among the studies. Most studies [20, 21, 23–25] chose to bisect each lymph node longitudinally for concurrent histopathological and OSNA testing. On the contrary, *Kazcka* et al. divided the lymph nodes into four parts in their first study [18], using alternating sections for OSNA and histopathological analysis, while in 2016 [22] cut lymph nodes into 2 mm intervals, assigning non-adjacent sections to OSNA or histological examination.

**Table 1:** Characteristics of included studies

Author	Year	Nation	Reference method	Nº patients	Nº lymph node	Tp	Tn	Fp	Fn	Discordance results (%)
Kaczka (19)	2014	Poland	HE	32	92	15	73	3	4	7 (7.6)
González (20)	2015	Spain	HE/IC	5	50	19	26	3	2	5 (10.0)
del Carmen (21)	2016	Spain	HE/IHC	37	284	75	160	18	14	32 (12.0)
Kaczka (22)	2017	Poland	HE	43	65	17	40	5	3	8 (7.7)
Iglesias Felip (23)	2019	Spain	HE/IC	35	470	50	251	57	2	59 (16.4)
Medas (24)	2019	Italy	HE/IHC	13	26	7	17	1	1	2 (7.7)
Iglesias (25)	2021	Spain	IC	42	572	120	380	67	5	72 (12.60)

*HE *Hematoxylin and Eosin staining, *IC* Imprint cytology, *IHC* Immunohistochemistry, *Tp* True positive, *Tn* True negative, *Fp* False positive, *Fn* False negative

Regarding the histopathological techniques employed, *Kazcka* et al. applied only Hematoxylin and Eosin (HE) staining in both studies [19, 22]. The rest of investigator groups, chose a combination of HE staining and IHC [20, 23] or HE staining alongside imprint cytology (IC) [20, 23, 25]. The OSNA RD-100i system by Sysmex Corp., Japan, was consistently used across all studies, with a general threshold for declaring positive lymph node metastasis set at ≥ 250 copies/ml of CK19 mRNA. The exact number of mRNA copies was carefully recorded to provide a detailed assessment of the metastatic burden. Additionally, only 4 groups [20, 21, 23, 25] described performing preoperative immunohistochemical screening to confirm CK19 expression, thus verifying the suitability of OSNA for individual patient profiles.

### Risk of bias and quality assessment

**Fig. 2:**
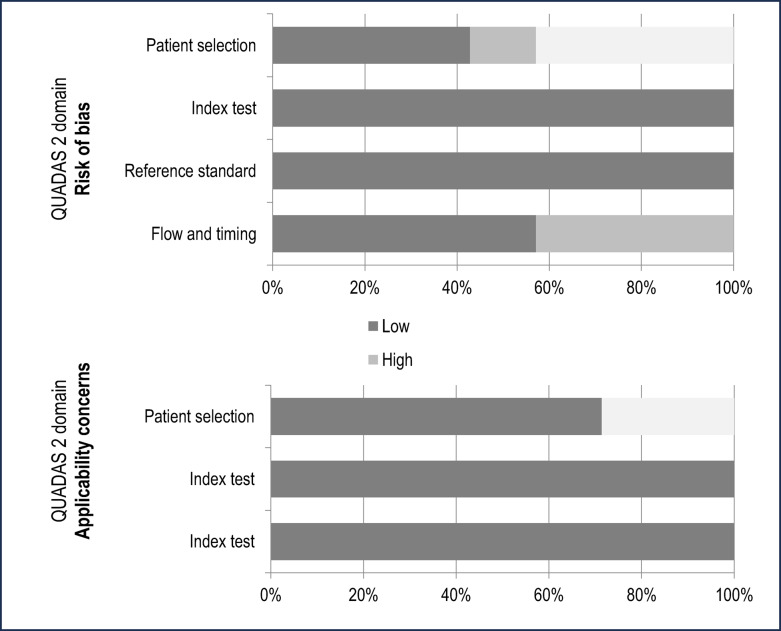
QUADAS-2 Domain proportion analysis. Proportion of studies with low, high, and unclear bias and applicability concerns, respectively

Each study’s quality was evaluated utilizing the QUADAS-2 criteria. The bias risk was found to be low, and the overall quality of the studies included ranged from moderate to high. QUADAS-2 evaluation of each study, as well as global proportion of studies with low, high or unclear concerns regarding applicability and risk of bias are detailed in Table 2; Fig. 2, respectively.

**Table 2:**
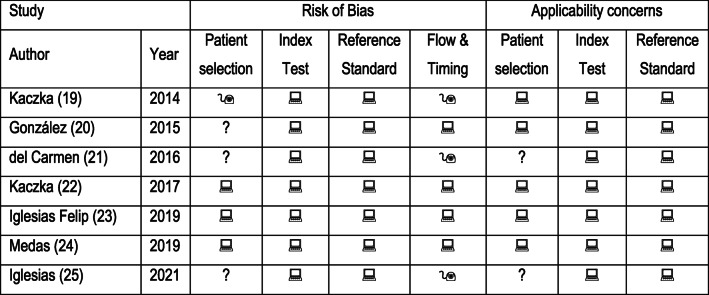
QUADAS-2 domains evaluation of each study

= low risk;  = high risk;? = unclear risk

### Diagnostic performance (Synthesis Results)

The Spearman’s correlation coefficient showed a strong negative correlation (*r* = − 0.821) with a p-value of 0.023, indicating a significant threshold effect in the data. This suggests an inverse relationship between sensitivity and specificity across the studies, reflecting variability in the diagnostic threshold applied across the included studies. The Cochran’s Q statistic was significantly high (Q = 138.21) with a p-value less than 0.001, and the I² statistic was 95.66%, indicating substantial heterogeneity among the studies. Given this significant heterogeneity, and considering that the included studies were conducted in different populations and settings, a random-effects model was employed for the meta-analysis to appropriately account for the variability observed across the studies and the potential differences in the populations included.

**Fig. 3:**
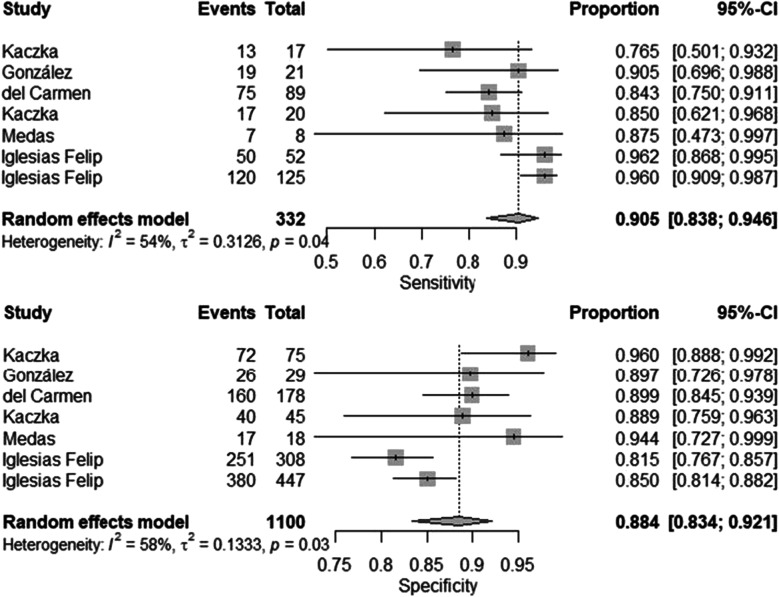
Forest plots of pooled sensitivity and specificity

**Fig. 4:**
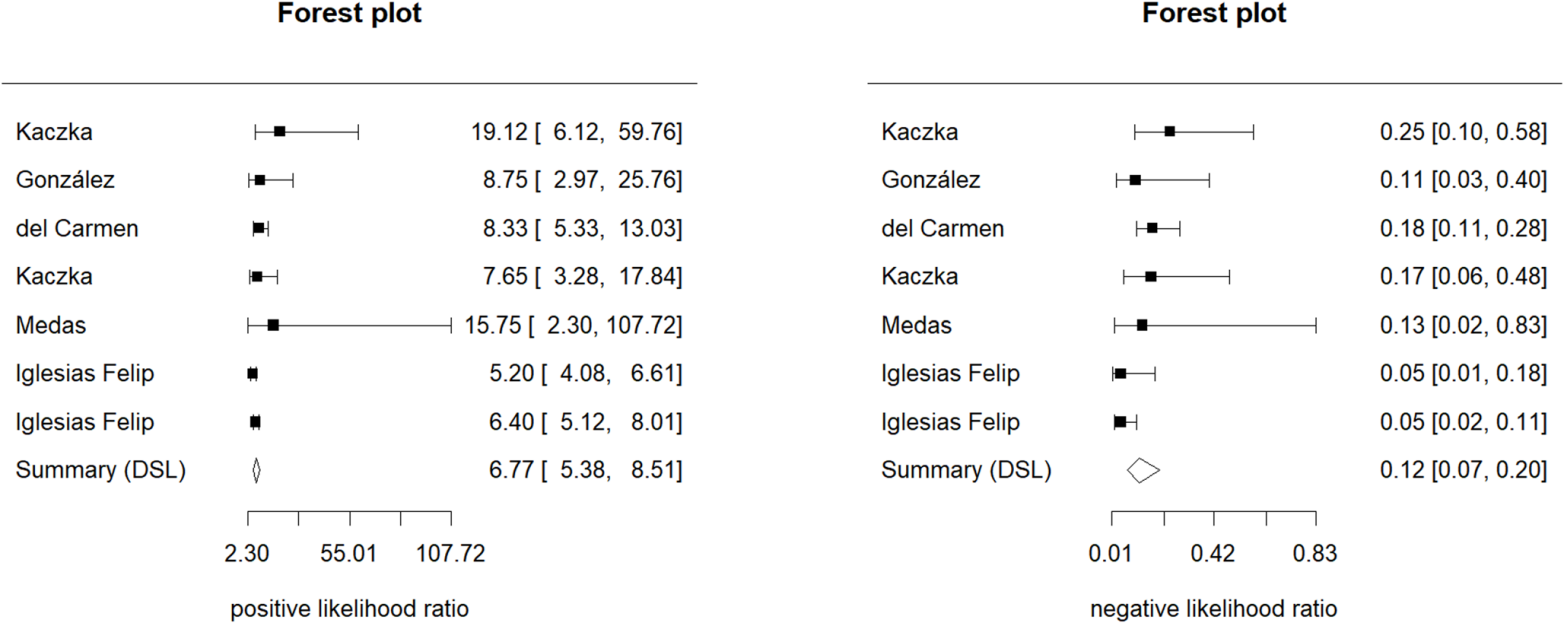
Forest plots of pooled positive and negative likelihood ratios

**Fig. 5:**
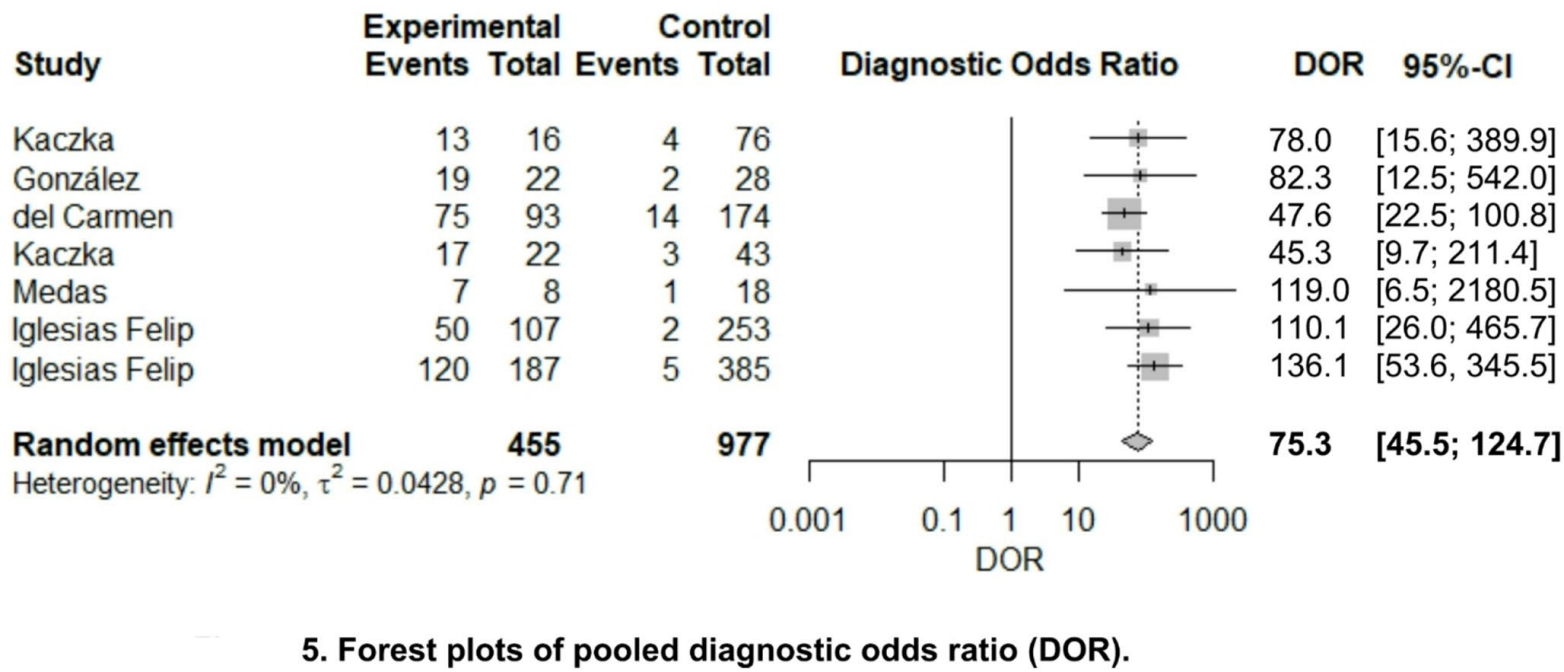
Forest plots of pooled diagnostic odds ratio (DOR)

**Fig. 6:**
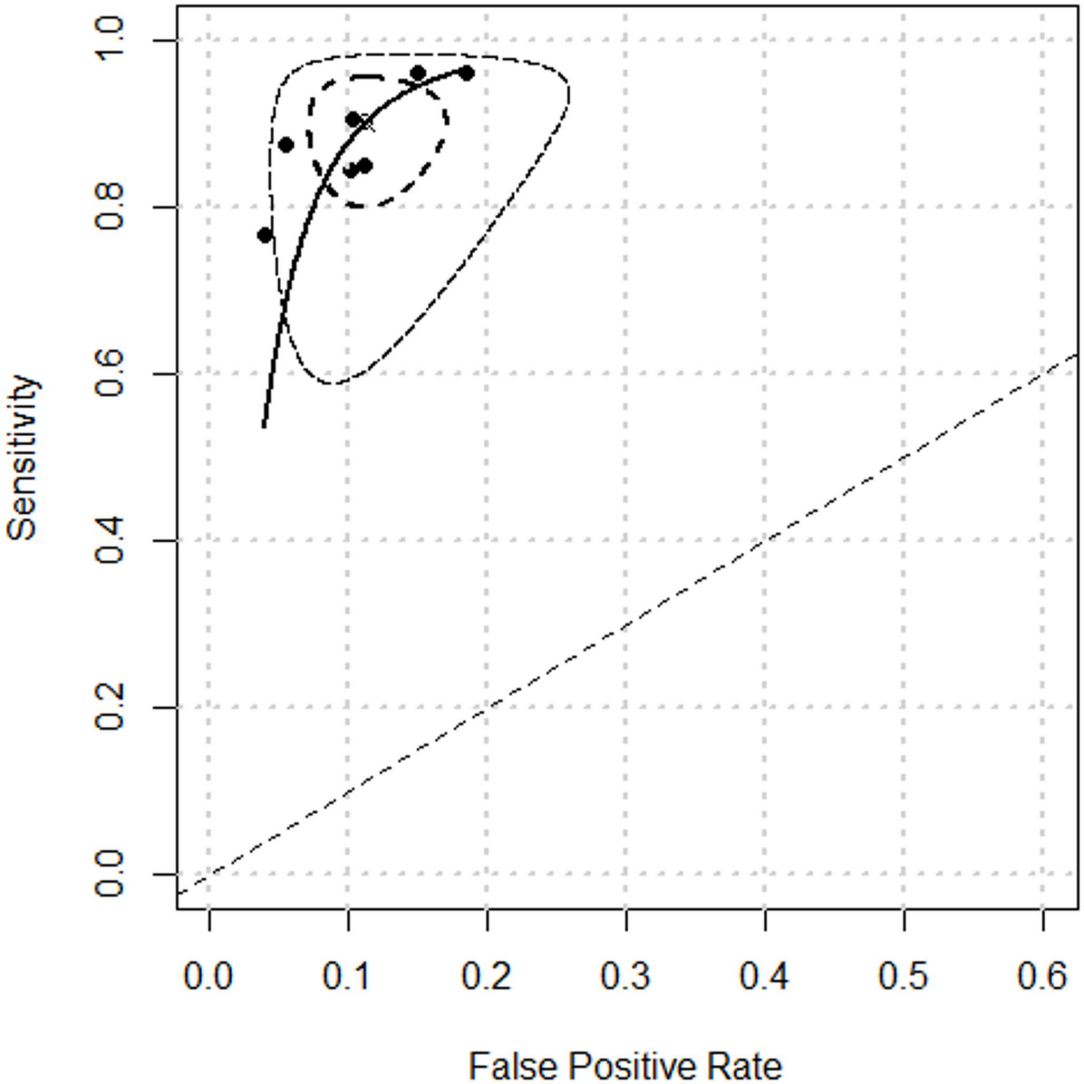
Summary receiver operating characteristics (SROC) curve

Figure 3 represent the results of pooled sensitivity, 0.905 (95% CI 0.838–0.946), and pooled specificity, 0.884 (95% CI 0.834–0.921), of OSNA for the detection of LNM. Regarding pooled positive likelihood ratio and negative likelihood ratio (Fig. 4), analysis resulted 6.77 (95% CI 5.38–8.51) and 0.12 (95% CI 0.07–0.20), respectively. Finally, pooled DOR (Fig. 5) was calculated resulting on 75.351 (95% CI 45.504–124.774) and AUC (Fig. 6) 0.848.

## Discussion

While OSNA has already become a protocolized tool in the evaluation of LNM of several carcinoma stirp tumors, such as breast cancer [26], its application in PTC is still in the early stages of development, being pioneered by some small sample studies [19–25]. In this meta-analysis, we evaluated the seven studies published about the LNM assessment with OSNA in PTC. Our results underscore high pooled sensitivity, pooled specificity and area AUC of OSNA for detecting LNM, which were 0.905 (95% CI 0.838–0.946), 0.884 (95% CI 0.834–0.921), and 0.848, respectively. The observed strong negative Spearman’s correlation (*r* = − 0.821) between sensitivity and specificity highlights a significant threshold effect, suggesting that OSNA’s superior sensitivity might detect true positives missed by standard methods. This phenomenon calls for a reevaluation of “false positives” in OSNA analyses, indicating that these may actually represent undetected metastases by conventional diagnostics. This finding suggests a need to reconsider the diagnostic thresholds in LNM detection for PTC.

Divergent guidelines underscore an ongoing controversy in the LNM assessment in PTC. Oriental guidelines [27–29] advocate for systematic prophylactic central compartment neck dissection, based on studies showing that over 30% of early-stage patients harbor occult LNM [5, 30], suggesting that omitting this procedure could lead to under-staging. On the other hand, occidental guidelines [4, 31] recommend to limit central lymph node dissection only for advanced tumors and performed by experienced surgeons, as this surgical procedure is not exempt of comorbidities such as 17.6% of transient and 2.4% permanent hypoparathyroidism, and 15.3% transient and 6.5% permanent recurrent laryngeal nerve injury [30, 32] and there is no clear evidence on the clinical impact in low-risk tumors. The SLNB, a cornerstone in various oncologic protocols [33], promised a solution by providing an intraoperative roadmap for or against lymph node dissection. However, its utility has been limited by false-negative rates [8], particularly related to the difficulty in detecting micrometastases by frozen section and/or imprint cytology examination, without immunohistochemical methods, especially during the surgery.

As demonstrated in breast cancer [26], the introduction of OSNA has emerged as a game-changer that can potentially outpace traditional methods with its turnaround processing time and semi-quantitative analysis, that excels in detecting even micrometastatic and small volume nodal disease. Notably, OSNA provides a rapid turnaround time of less than 40 min and can simultaneously evaluate up to four lymph nodes [34], in contrast to pathological evaluations which typically assess one node at a time. This efficiency is critical in meeting the demands for quick intraoperative diagnostic processes and allows for immediate surgical decision-making, as remarked Kaczka [22] and Iglesias [23]. On the other hand, OSNA’s methodology is objective and not dependent on the subjective interpretations that characterize traditional histopathology, but it quantitatively measures CK19 mRNA copies with the amount directly reflecting the presence of cancer cells within the nodes [34]. This process adheres to a unified protocol, maintaining a consistent threshold of 250 CK19 mRNA copies/ml across all studies [19–25] which supports the uniformity and reproducibility of the results. Our findings corroborate the high diagnostic accuracy (AUC = 0.848) of OSNA in detecting LNM in PTC, aligning with previous research and underscoring its reliability [18, 20, 21]. Given this high level of accuracy, OSNA proves to be a sufficiently robust tool for the management of PTC, enhancing the precision of intraoperative evaluations and potentially improving patient outcomes.

Despite the robustness of OSNA technique across the validation studies analyzed, discrepancies remain due to methodological differences. Most studies typically split the lymph nodes into halves or quarters, analyzing one portion with conventional methods and the other with OSNA, which may introduce sampling bias. However, the methodology proposed by Iglesias [25] uniquely allows for the complete analysis of the lymph node using OSNA alongside conventional control via cytological imprint, potentially providing a more comprehensive evaluation. On the other hand, while OSNA provides semi-quantitative values, studies by González [20] and del Carmen [21] have not shown statistically significant correlations between the number of CK19 mRNA copies and the size and weight of the nodes. In contrast, Iglesias’ study [25], which includes a larger patient cohort, did find a statistically significant positive correlation, suggesting a potential relationship between tumor burden and mRNA expression. However, these correlations could not be thoroughly evaluated in the meta-analysis due to insufficient data across the studies.

Several inherent limitations of OSNA also need consideration. Up to 30% of PTCs are CK19 negative [35], requiring the preoperative validation of CK19 expression in the primary tumor before employing OSNA. This preoperative evaluation is not reported in all analyzed studies [19, 22, 24]. Furthermore, OSNA cannot perform morphological analysis of nodal metastases or extranodal extension, which limits its diagnostic scope. Lastly, the clinical significance of performing central lymph node dissection in the presence of micrometastases detected by OSNA remains unclear, highlights the need for further clinical evidence to define the oncological implications of such findings.

Our meta-analysis has several limitations that warrant consideration. First, the inherent heterogeneity among the included studies can challenge the applicability of our findings across different clinical settings. Secondly, while we adhered to PRISMA guidelines, certain analyses were not feasible due to the limited number of included studies. Specifically, a sensitivity analysis was not performed because the number of studies available restricted the robustness of such an analysis. Moreover, the limited number of studies (*n* = 7) and inconsistent data reporting hindered the ability to conduct meaningful subgroup analyses. Furthermore, publication bias was not assessed using a funnel plot, as it is generally recommended only when at least 10 studies are included in a meta-analysis [36]. Thirdly, the scope of our analysis was restricted to studies published in English and Spanish, potentially omitting relevant research conducted in other languages. This language limitation may lead to publication bias, as pivotal studies with negative or neutral results often published in non-English journals could be excluded. Lastly, the absence of randomized controlled trials (RCTs) among the studies reviewed limits the strength of our conclusions. The studies included are predominantly observational, which are more susceptible to selection bias and other confounding factors that RCTs are designed to mitigate. This lack of higher-quality evidence highlights the need for future research to incorporate RCTs to better ascertain the efficacy and reliability of OSNA in clinical practice.

In conclusion, this meta-analysis provides evidence supporting the diagnostic accuracy of OSNA compared to conventional histopathological techniques, indicating its potential utility for surgical teams aiming to refine the precision of SLNB. Nevertheless, the clinical implementation of OSNA should be undertaken with caution, as its widespread adoption must be substantiated by further randomized controlled trials that assess long-term outcomes and clearly demonstrate an improvement in patient survival and overall management.

## Supplementary Information

Below is the link to the electronic supplementary material.

Supplementary file1 (DOCX 32.9 KB)

## Data Availability

No datasets were generated or analysed during the current study.
